# Metabolite Profiles of Red and Yellow Watermelon (*Citrullus lanatus*) Cultivars Using a ^1^H-NMR Metabolomics Approach

**DOI:** 10.3390/molecules25143235

**Published:** 2020-07-15

**Authors:** Fadzil Sulaiman, Amalina Ahmad Azam, Muhammad Safwan Ahamad Bustamam, Sharida Fakurazi, Faridah Abas, Yee Xuan Lee, Atira Adriana Ismail, Siti Munirah Mohd Faudzi, Intan Safinar Ismail

**Affiliations:** 1 Laboratory of Natural Products, Institute of Bioscience, Universiti Putra Malaysia, Serdang 43400, Malaysia; mfadzilsulaiman90@gmail.com (F.S.); amalina_azam@hotmail.com (A.A.A.); safwan.upm@gmail.com (M.S.A.B.); faridah_abas@upm.edu.my (F.A.); leeyeexuan0613@gmail.com (Y.X.L.); atiradriana@gmail.com (A.A.I.); sitimunirah@upm.edu.my (S.M.M.F.); 2 Laboratory of Vaccines and Immunotherapeutics, Institute of Bioscience, Universiti Putra Malaysia, Serdang 43400, Malaysia; sharida@upm.edu.my

**Keywords:** ^1^H-NMR, Citrullus lanatus, varieties, discriminants, carotenoids

## Abstract

Watermelon, a widely commercialized fruit, is famous for its thirst-quenching property. The broad range of cultivars, which give rise to distinct color and taste, can be attributed to the differences in their chemical profile, especially that of the carotenoids and volatile compounds. In order to understand this distribution properly, water extracts of red and yellow watermelon pulps with predominantly polar metabolites were subjected to proton nuclear magnetic resonance (^1^H-NMR) analysis. Deuterium oxide (D_2_O) and deuterated chloroform (CDCl_3_) solvents were used to capture both polar and non-polar metabolites from the same sample. Thirty-six metabolites, of which six are carotenoids, were identified from the extracts. The clustering of the compounds was determined using unsupervised principal component analysis (PCA) and further grouping was achieved using supervised orthogonal partial least squares discriminant analysis (OPLS-DA). The presence of lycopene, *β*-carotene, lutein, and prolycopene in the red watermelon plays an important role in its differentiation from the yellow cultivar. A marked difference in metabolite distribution was observed between the NMR solvents used as evidenced from the PCA model. OPLS-DA and relative quantification of the metabolites, on the other hand, helped in uncovering the discriminating metabolites of the red and yellow watermelon cultivars from the same solvent system.

## 1. Introduction

Chemometrics is a tool which utilizes mathematical and statistical models in gathering information from a chemical system. With the advancement of computer science, raw chemical data can be remodeled to derive patterns or new variables to address problems in the field of pharmaceutical science [1], biochemistry [2], medicine [3], natural product research [4], and agriculture [5]. This approach helps in the handling of large sets of data such as those of the omics in understanding a biological system or process. 

The visualization and clustering of spectral input by means of multivariate data analysis (MVDA) assist in the assessment of biochemical processes of a complex set of phenotypes [6]. In the metabolomics analysis of a plant, the effect of a particular environment or stressor on a species can be studied by the qualitative or quantitative measurement of its metabolites, such as those presented in the study of *Nicotiana tabacum* and *Brassica rapa* (L.) against infection and herbivory, respectively [7,8]. Integrative taxonomy, which incorporates metabolomics, has been suggested in overcoming the limitations of morphological and molecular classification of plants [9]. Interest in metabolome diversity of different cultivars of the same plant species is also gaining traction for the wealth of information it provides, as evidenced in the study of two *Oryza sativa* L. [10] and 43 *Camellia sinensis* L. cultivars [11].

The general goal of metabolomics studies in profiling and classifying plant metabolites by way of fingerprinting can be achieved by incorporating proton nuclear magnetic resonance (^1^H-NMR) analysis. This spectroscopic method allows the simultaneous identification of primary and species-specific secondary metabolites while permitting the comparison of their concentrations without the need of an individual calibration curve [12]. Having a general view of the metabolites with different polarity, behavior, and stability in just one experiment is advantageous in studies concerning the interaction between the phenotypes with the environment, although large sets of data are needed to ensure its reproducibility [13]. Other possible shortcomings of this approach, such as the limitation in metabolite identification from signal overlapping, are rapidly overcome with the incorporation of two-dimensional NMR. As for low sensitivity despite upgrades in the hardware used, approaches that optimize samples and pulse sequences, improve the electronics of the probe and coils, and increase the magnetic fields have been suggested to resolve this issue [14]. The interpretation and the search for new variables from large sets of ^1^H-NMR data can be accomplished using statistical interpretation such as principal component analysis (PCA), a technique which reduces data dimensionality while minimizing information loss [15]. Meanwhile, in the case of data grouping within a group, a supervised statistical method such as orthogonal partial least squares discriminant analysis (OPLS-DA) with a validation method can be utilized [16]. 

Watermelon or *Citrullus lanatus* (Thunb.) is a vine-like flowering plant belonging to the Cucurbitaceae family. Originating from the African continent, this fruit has been widely cultivated and is highly favored for its sweet and refreshing taste. Studies on the bioactivities have revealed that the presence of cucurbitacin E isolated from the pulp displayed a good anti-inflammatory activity through the inhibition of cyclooxygenase (COX) and reactive nitrogen species (RNS), whereas lycopene, β-carotene, and ascorbic acid contributed greatly to the fruit’s antioxidant property [17,18]. Because interest in the health benefits of carotenoids has grown, it is worthwhile to look into watermelon cultivars that possess a wide range of this phytochemical group [19,20]. Aside from the reasons stated above, the decision in using a metabolomics approach was made since limited studies are available for this particular species. One study highlights the sugar and amino acid concentrations between two watermelon varieties and another explores the sensory quality between a watermelon grafted onto a gourd and onto pumpkin rootstocks [21,22]. Both studies did not report on the carotenoid profile. 

Therefore, the aim of this study was to outline the metabolites present in the red and yellow watermelon cultivars via a metabolomics approach, with a special interest on the carotenoid group. Based on the color of the flesh, it can be deduced that both cultivars may give a different set of phytochemicals responsible for their pigments. However, the interaction between different groups of metabolites can also be conceptualized using a more analytical approach. The results obtained from this study can be used to better understand the biochemical process of this fruit in addition to the advantage of correlating the identified metabolites to their medicinal values to tailor one’s dietary intake.

## 2. Results and Discussion

Carotenoids, known as tetraterpenes, are a group of natural products with a carbon-40 skeleton. These phytochemicals give a strong pigment as a result of their highly conjugated carbons, and the color produced is known to have a protective effect against ultraviolet (UV) rays and free radicals [23]. The reported carotenoids in the red watermelon are predominantly lycopene with trace amounts of phytoene, phytofluene, *ζ*-carotene, α-carotene, lutein, zeaxanthin, and violaxanthin, whereas those of the yellow watermelon are neoxanthin, violaxanthin, and neochrome [24,25,26]. Other common metabolite groups that are found in watermelons include sugars (glucose, sucrose, and fructose), amino acids (isoleucine, valine, citrulline, arginine, and glutamine), organic acids (malic acid, citric acid, quinic acid, and tartaric acid), and a set of volatile compounds [22,25]. As for its nutritional value, a 100 g portion of watermelon contains about 91% water, 6% sugar and 0.4% fiber. Potassium, phosphorus, magnesium, calcium, and ascorbic acid are among the micronutrients which are abundant in the fruit, making it a healthy dietary choice [27]. Due to the lack of ^1^H-NMR data for the identification of metabolites in watermelons, profiling was done using Chenomx software (v.5.1, Edmonton, AB, Canada) and by comparing the chemical shifts of the spectra with the ones from previously reported literature [28,29,30].

### 2.1. H-NMR of the Pulp Extracts of Red and Yellow Watermelon Cultivars

In this research, the extraction of watermelon pulp was done using Millipore water to mimic dietary intake. Ultrasonication was chosen for the extraction technique as this maximized the yield of lycopene as shown in a study involving tomatoes [31]. This may be true for other metabolites as well, for the agitation and cell wall breakdown help to release the metabolites from their cellular condition. Carotenoids can be divided into non-oxygenated carotenes and oxygenated xantophylls, the former being more orange in color while the latter is yellow. Both exist abundantly in an average diet of a person, especially from the fruits and vegetables. Due to the metabolites’ lipophilic nature, the water extract was subjected to NMR analysis using two types of solvents, namely deuterium oxide (D_2_O) and deuterated chloroform (CDCl_3_), to capture the metabolites present in both extremes of the polarity scale. A total of 36 metabolites were identified from the samples as shown in Table 1, of which 27 are from the D_2_O solvent and the remaining 9 from CDCl_3_. Compounds such as methionine and lysine, from a previously reported metabolomics study of watermelons, were not able to be profiled [21]. This is probably due to the difference in cultivar types [21], harvest time [32], sample processing method [33], or even from the overlapping of signals in the ^1^H-NMR spectra [13].The same applies to the unidentified carotenoids such as phytoene, phytofluene, ζ-carotene, α-carotene, neoxanthin, and neochrome, which were reported in other studies [24,25,26]. Despite the limitations in the detection of some metabolites, no metabolomics study has highlighted the color difference of the watermelon cultivars’ flesh as presented in the current report.

**Figure 1:**
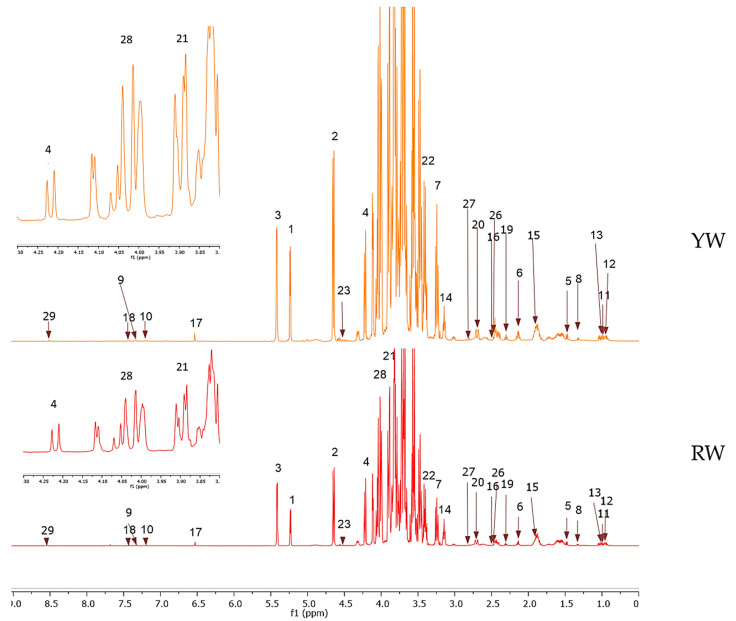
Representative ^1^H-NMR spectra of red watermelon (RW) and yellow watermelon (YW) in deuterium oxide (D_2_O); inset: 10× expansion of 3.80 to 4.80 ppm. Identified metabolites: (1) α-Glucose, (2) β-Glucose, (3) Sucrose, (4) Fructose, (5) Alanine, (6) Glutamine, (7) Arginine, (8) Threonine, (9) Tryptophan, (10) Tyrosine, (11) Valine, (12) Leucine, (13) Isoleucine, (14) Citrulline, (15) Acetate, (16) Citrate, (17) Fumarate, (18) Phenylalanine, (19) 4-Aminobutyrate, (20) Malic acid, (21) Aspartate, (22) Xylose, (23) Ascorbic acid, (26) Succinic acid, (27) Aspartic acid, (28) Cucurbitacin E, (29) Formic acid.

**Figure 2:**
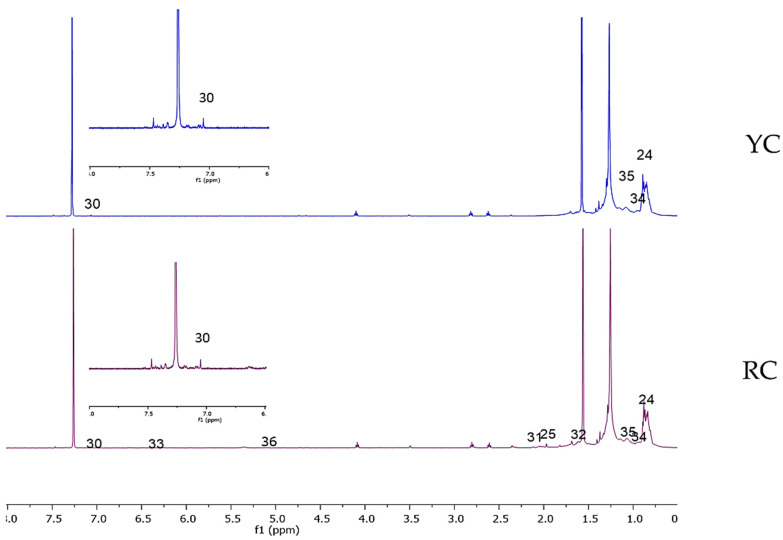
Representative ^1^H-NMR spectra of red watermelon (RC) and yellow watermelon (YC) in deuterated chloroform (CDCl_3_); inset: 2.5× expansion of the aromatic region from 6.50 to 8.00 ppm. Identified metabolites: (24) Linoleic acid, (25) Acetic acid, (30) Gallic acid, (31) Lycopene, (32) β-Carotene, (33) Lutein, (34) Violaxanthin, (35) Zeaxanthin, (36) Prolycopene.

The representative ^1^H-NMR spectra for each sample are presented in Figure 1 and Figure 2 (Figure S1). No notable difference is observed between the RW and YW samples except for the intensity of the peaks, which suggests the varying concentrations of the metabolites. A marked overlapping of signals is observed between 3.25 and 4.25 ppm, which limits the identification of metabolites from this region for both samples. The peaks of the sugars dominate the spectra and can be clearly seen between the region of 4.5–5.5 ppm. As for the CDCl_3_ samples, a marked difference is present in the region of 1.7–2.5 ppm, which explains the identification of acetic acid and lycopene peaks exclusively from the RC sample. However, not many peaks were identified in the low-field region, which is characteristic of the carotenoid group; this may be due to the principle of liquid–liquid extraction in which limited non-polar compounds are drawn out using the CDCl_3_ solvent from the predominantly polar group of metabolites concentrated in the water extracts. Multivariate data analysis using SIMCA-P software (v.14.1, Umetrics, Umeȧ, Sweden) was performed to obtain the principal component analysis (PCA) model to better understand the metabolite variation within the different groups.

### 2.2. Multivariate Data Analysis of the Pulp Extracts of Red and Yellow Watermelon Cultivars

**Figure 3:**
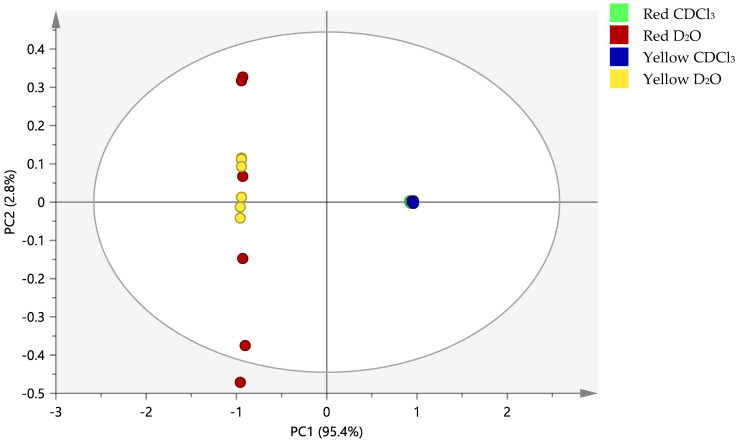
The principal component analysis (PCA) score plot of the ^1^H-NMR data representing red and yellow watermelon cultivars in two different solvents (D_2_O and CDCl_3_); R2X = 0.991, Q2X = 0.983, *n* = 6.

Figure 3 shows the clustering based on the ^1^H-NMR data of the red and yellow watermelon cultivars analyzed in the D_2_O and CDCl_3_ solvents. Satisfactory goodness of fit and predictability are observed from the values of R2X and Q2X, which are 0.991 and 0.983, respectively. This follows the criteria of a good model, i.e., Q2 > 0.5, R2 > Q2, and the difference between both values is between 0.2 to 0.3 [34]. A variation total of 98.2% is shown by the first two principal components with PC1 accounting for 95.4% and PC2 for 2.8%. This gives rise to a good separation of the solvent groups by PC1, whereas the different watermelon cultivars extracted by the same solvent system, particularly CDCl_3_, seem to be clustered together. One distinct outlier is identified from the RW group; however, the data are included in the plot as its DModX value (Figure S2) is not twice as large as the maximum tolerable distance (Dcrit) value to be considered a moderate outlier. This was confirmed with its Hotelling’s T2 value (Figure S3), which is lower than the confidence limits [34].

**Figure 4:**
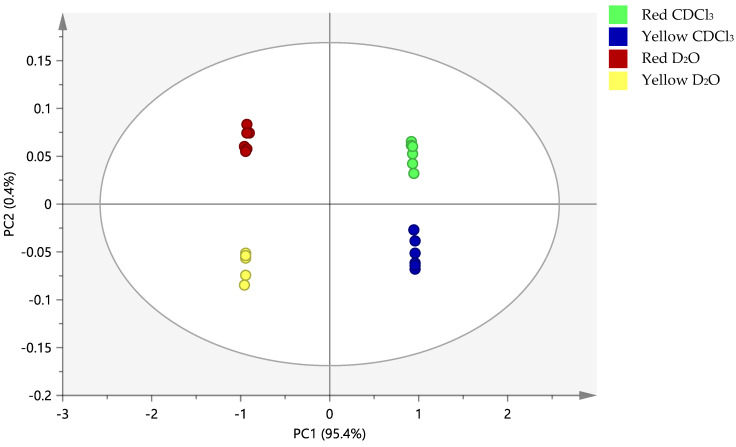
The orthogonal partial least squares discriminant analysis (OPLS-DA) score plot of the ^1^H-NMR data representing red and yellow watermelon cultivars in two different solvents (D_2_O and CDCl_3_); R2X = 0.995, Q2X = 0.862, *n* = 6.

PCA is often useful in exposing separation when the variation between the class is greater than within the class [16]. Where interest in both groups of variables is of concern, it is best to adopt a supervised modelling approach using orthogonal partial least squares discriminant analysis (OPLS-DA). Figure 4 shows the OPLS-DA performed on the ^1^H-NMR data of the red and yellow watermelon cultivars in the D_2_O and CDCl_3_ solvents (Figure S4 and S5); the data from both the solvent and cultivar groups are well separated. Satisfactory goodness of fit and predictability of this model are observed from the values of R2X and Q2X, which are 0.995 and 0.862, respectively [34]. The 100-permutation test and CV-ANOVA validation methods were used to nullify the risk of overfitting, which comes with a supervised statistical analysis. In the permutation test (Figure S6–S9), a model with a Y-axis intercept below 0.3 for R2 and 0.05 for Q2 and a non-horizontal R2 line is said to be reliable. These values are apparent for the 4 classes of data tested except for their R2 value, which is slightly above 0.3. The CV-ANOVA test (Table S1), on the other hand, confirmed that the model is of optimum fit with the value of 1.22 × 10^−9^, which is lower than the cut-off of 0.05 [34].

The OPLS-DA loading column plot, however, does not give rise to significant discriminated metabolites as the error bar for all of the columns crosses the X-axis. Nevertheless, the model predicted that higher concentrations of tryptophan, fumarate, α-glucose, β-glucose, malic acid, xylose, arginine, 4-aminobutyrate, glutamine, citrulline, and threonine in RW and phenylalanine, tyrosine, sucrose, fructose, cucurbitacin E, aspartate, leucine, aspartic acid, and alanine in YW differentiate both groups from one another. Meanwhile, higher concentrations of gallic acid, linoleic acid, violaxanthin, and zeaxanthin in YC differentiate this group from the RC group. Relative quantification was carried out to further analyze this aspect. 

### 2.3. Relative Quantification

Relative concentrations of metabolites with the variable importance in projection (VIP) value of more than 1 (Figure S10), which indicates those which have the most discriminatory attributes [35], was determined from the OPLS-DA. This was done to single out the metabolites responsible for class separation within the solvent group, which was not achieved using the PCA and OPLS-DA models. The mean peak area of characteristic ^1^H-NMR signals was chosen, and the chemical shifts selected included phenylalanine at 7.42 ppm, tryptophan at 7.3 ppm, tyrosine at 6.9 ppm, fumarate at 6.54 ppm, sucrose at 5.42 ppm, α-glucose at 5.22 ppm, β-glucose at 4.62 ppm, malic acid at 4.34 ppm, fructose at 4.22 ppm, cucurbitacin E at 4.02 ppm, aspartate at 3.9 ppm, leucine at 3.7 ppm, xylose at 3.38 ppm, arginine at 3.22 ppm, aspartic acid at 2.66 ppm, 4-aminobutyrate at 2.3 ppm, glutamine at 2.14 ppm, citrulline at 1.58 ppm, alanine at 1.46 ppm, threonine at 1.34 ppm, gallic acid at 7.06 ppm, linoleic acid at 1.18 ppm, violaxanthin at 1.14 ppm, and zeaxanthin at 1.78 ppm. 

**Figure 5:**
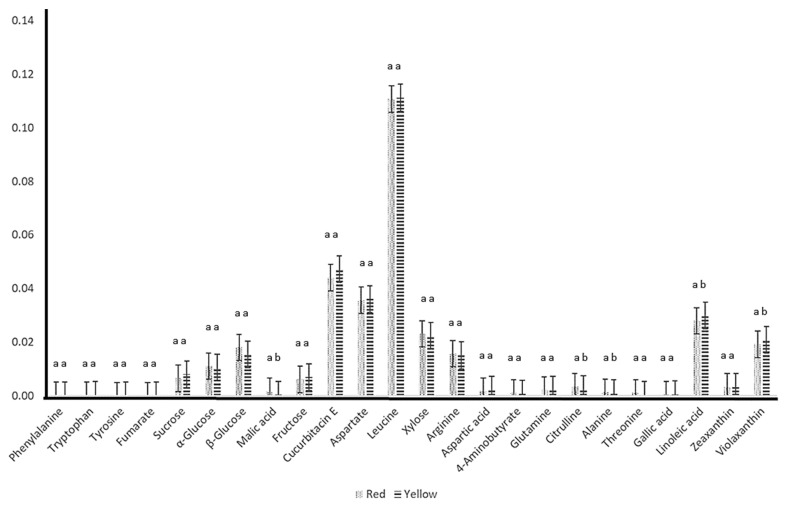
Relative quantification of the discriminatory metabolites identified from the yellow and red watermelon cultivars in D_2_O and CDCl_3_ solvents based on the mean peak area of the ^1^H-NMR signals. The different letters (a and b) indicate significant difference (*p* < 0.05) between the two cultivars.

The results, as shown in Figure 5, revealed that RW has higher concentrations of fumarate, α-glucose, β-glucose, malic acid, xylose, arginine, 4-aminobutyrate, citrulline, alanine, and threonine, whereas YW has higher concentrations of phenylalanine, tryptophan, tyrosine, sucrose, fructose, cucurbitacin E, aspartate, leucine, aspartic acid, and glutamine. YC has higher relative concentrations of gallic acid, linoleic acid, violaxanthin, and zeaxanthin as compared to those in RC. Overall, the data closely resemble those predicted in the OPLS-DA loading column model, although only malic acid, citrulline, alanine, linoleic acid, and violaxanthin showed significant difference (*p* < 0.05) between both cultivars, as proven by the one-way ANOVA test. 

This study confirms the presence of widely studied phytoconstituents from the *Citrullus lanatus* species that are deemed as medicinally beneficial, such as lycopene, cucurbitacin E, citrulline, arginine, and ascorbic acid, among other metabolites [17,36,37]. Of these, lycopene and citrulline can be said to contribute significantly to setting the red watermelon cultivar apart from the yellow cultivar using a metabolomics approach, eventually making it a healthier dietary choice. However, the synergistic interaction between the metabolites must be taken into consideration as this can affect the bioavailability of the phytochemicals after ingestion and, therefore, influence their efficiency [38,39]. The use of less polar solvents for extraction coupled with other spectrometry tools (e.g., liquid chromatography–mass spectroscopy (LC–MS), gas chromatography–mass spectroscopy (GC–MS) and infrared spectroscopy) can be employed to improve the identification of less polar metabolites, such as the carotenoids and volatile compounds from both cultivars.

## 3. Materials and Methods 

### 3.1. Chemicals

Deuterium oxide (D_2_O, 99.9%), deuterated chloroform (CDCl_3_, 99.8%) with 0.03% tetramethylsilane (TMS), sodium deuterium oxide (NaOD), and potassium dihydrogenphosphate (KH_2_PO_4_) were purchased from Merck (Darmstadt, Germany). 3-(Trimethylsilyl)propionic acid-d4 sodium salt (TSP) was obtained from Sigma-Aldrich (St. Louis, MO, USA).

### 3.2. Plant Material

The cultivation of red and yellow watermelons (*Citrullus lanatus* (Thunb.) Matsum. et Nakai) was carried out from March to May 2018 in Taman Pertanian Universiti (TPU: 2.986768, 101.70932) Universiti Putra Malaysia (UPM). The seeds were of F1 hybrid and procured from a local company (Green World Genetics Snd. Bhd.). The red watermelon is of the 310 variety (Red Rocky), while the yellow watermelon is of the 393 variety (Gold Dragon). The plants were grown organically; no pesticide was used at all growth stages and a combination of chopped grass and kale, animal waste, and earthworms was provided as fertilizer. Six each of the red and yellow watermelons were harvested at three months of age between 22 and 28 May 2018 and stored in a −80 °C freezer immediately after harvested. On the processing day, the watermelon pulp was scooped out an inch away from the rind after the fruit was thawed to room temperature. The pooled pulp was deseeded and crushed to draw out the juice before it was subjected to continuous freeze-drying (Labconco, Kansas City, MO, USA) for three days. The powdered watermelon biomass was kept in a tightly sealed amber jar and stored in −80 °C until further use.

### 3.3. Extraction

Watermelon water extract was prepared using the ultrasonication method [40,41]. Powdered biomass (10 g each) was weighed and dissolved in 200 mL Millipore water. The solution was subjected to sonication for 1 h at a temperature below 40 °C. The extract was filtered using muslin cloth, and the residual pulp was extracted a second time using fresh Millipore water. The filtrate was pooled and freeze-dried (Labconco) to powder form before it was stored in a −80 °C freezer. This process was repeated for all red and yellow watermelons, and the yield and water loss were calculated as shown in Table 2.

### 3.4. NMR Analysis

This method follows that outlined by the authors of [12] with some modifications. KH_2_PO_4_ buffer (0.1M) and an internal standard of 0.01% TSP were added to the D_2_O solvent, and the pH of the mixture was adjusted to 6.0 by carefully adding NaOD. Powdered extract (50 mg) was dissolved in 1 mL of the buffered D_2_O and vortexed for 30 s. The solution was sonicated in an ultrasonic bath for 5 min at a temperature below 40 °C followed by centrifugation at 13,000 rpm for 10 min. The supernatant of 600 µL was transferred into a clean 5 mm NMR tube. The sample was subjected to ^1^H-NMR using D_2_O as an internal lock. This analysis was performed using a 500 MHz Varian INOVA NMR spectrometer (Varian Inc., Palo Alto, CA, USA) at a frequency of 499.887 MHz and a temperature maintained at 25 °C. The water peak was suppressed by applying a prestarvation (PRESAT) pulse sequence. The acquisition time of 3.53 min was required for 64 scans with a width of 20 ppm, a pulse width of 3.75 μs, and a relaxation delay of 1.0 s [42]. The internal standard (TSP) was calibrated to 0.00 ppm. This procedure was done on all 12 samples (6 each from the red and yellow watermelon water extracts) and later replicated using CDCl_3_ solvent with 0.03% TMS as an internal standard; the PRESAT step for this batch was omitted. The NMR analysis for each sample was done only once and a total of 24 spectra were obtained.

### 3.5. Data Processing and Statistical Tests

Microsoft Excel 2016 was used to perform the one-way analysis of variance (ANOVA) test at 95% confidence interval to determine the significant difference between the variables. Phasing, alignment, and baseline correction of all spectra were completed using Chenomx software (v.5.1, Edmonton, AB, Canada). The ACSII files of the chemical shifts in the range of δ 0.3–10.0 were binned at the spectral width of δ 0.04 for all 24 spectra. The residual water peak between the region of 4.81–4.95 ppm and the deuterated chloroform peak between the region of 7.25–7.27 ppm were excluded from this process. SIMCA-P software (v.14.1, Umetrics, Umeȧ, Sweden) with Pareto scaling was used to perform unsupervised principal component analysis (PCA) to better understand the similarities and differences of the metabolites from the two watermelon cultivars and the NMR solvents used. The model validation and significance were determined from the R2X and Q2X values. Supervised orthogonal partial least squares discriminant analysis (OPLS-DA) with 100-permutation and CV-ANOVA validation methods was used to further demarcate the groups.

## 4. Conclusions

The use of a chemometric tool is advantageous in studying closely related biological samples of the same species by means of metabolite distribution. This study proved the usefulness of ^1^H-NMR in the visualization of complex chemical data of two watermelon cultivars and their relatedness to the physical property of the fruits. By using metabolite profiling and MVDA, the red watermelon cultivar can be clustered apart from the yellow to determine the presence of lycopene and a high concentration of citrulline. The choice of two NMR solvents of different polarities was made to capture a wider group of metabolites from both polar and non-polar ends, although the latter, which harbors most of the carotenoids, is limited since the samples are derived from a more polar water extract. Future research on the incorporation of multiple spectrometric approaches with less polar solvents can be developed to streamline the metabolite identification step in order to better discriminate the two cultivars.

## Figures and Tables

**Table 1:** Identified metabolites in red and yellow watermelon cultivars using two types of nuclear magnetic resonance (NMR) solvent (R: red watermelon, Y: yellow watermelon, W: deuterium oxide, C: deuterated chloroform).

No.	Metabolite	Chemical Shift (Multiplicity)	RW	YW	RC	YC
1	α-Glucose	5.23 (d, *J* = 4.0 Hz)	+	+	-	-
2	*β*-Glucose	4.64 (d, *J* = 8.0 Hz)	+	+	-	-
3	Sucrose	5.41 (d, *J* = 4.0 Hz)	+	+	-	-
4	Fructose	4.22 (d, *J* = 9.0 Hz)	+	+	-	-
5	Alanine	1.48 (d, *J* = 7.5 Hz)	+	+	-	-
6	Glutamine	2.14 (m), 2.47 (m)	+	+	-	-
7	Arginine	3.76 (m), 1.89 (m), 1.63 (m), 3.24 (t, *J* = 9.0 Hz)	+	+	-	-
8	Threonine	1.33 (d, *J* = 7.0 Hz)	+	+	-	-
9	Tryptophan	7.20 (t, *J* = 7.0 Hz), 7.29 (t, *J* = 8.0 Hz), 7.32 (s), 7.54 (d, *J* = 8.0 Hz), 7.74 (d, *J* = 8.0 Hz)	+	+	-	-
10	Tyrosine	7.19 (t, *J* = 7.0 Hz), 6.90 (m)	+	+	-	-
11	Valine	0.99 (m), 1.04 (d, *J* = 7.0 Hz)	+	+	-	-
12	Leucine	3.72 (t, *J* = 4.0 Hz), 0.93 (d, *J* = 7.5 Hz), 0.96 (m)	+	+	-	-
13	Isoleucine	1.01 (m), 0.94 (m)	+	+	-	-
14	Citrulline	3.15 (t, *J* = 7.0 Hz), 1.58 (m), 3.76 (m)	+	+	-	-
15	Acetate	1.91 (s)	+	+	-	-
16	Citrate	2.50 (d, *J* = 8.0 Hz), 2.70 (dd, *J* = 15.5, 3.5 Hz)	+	+	-	-
17	Fumarate	6.53 (s)	+	+	-	-
18	Phenylalanine	7.33 (m), 7.39 (m), 7.43 (m)	+	+	-	-
19	4-Aminobutyrate	2.31 (t, *J* = 7.5 Hz), 3.01 (t, *J* = 7.5 Hz)	+	+	-	-
20	Malic acid	4.32 (dd, *J* = 10.0, 3.5 Hz), 2.70 (dd, *J* = 15.5, 3.5 Hz), 2.82 (dd, *J* = 18.0, 4.5 Hz)	+	+	-	-
21	Aspartate	2.70 (dd, *J* = 15.5, 3.5 Hz), 2.82 (dd, 18.0, 4.5 Hz), 3.90 (dd, *J* = 10.5, 3.5 Hz)	+	+	-	-
22	Xylose	3.39 (t, *J* = 9.5), 4.54 (d, *J* = 19.5 Hz), 5.19 (m)	+	+	-	-
23	Ascorbic acid	4.54 (d, *J* = 19.5 Hz)	+	+	-	-
24	Linoleic acid	0.88 (m), 1.17 (m)	-	-	+	+
25	Acetic acid	1.97 (s)	-	-	+	-
26	Succinic acid	2.48 (s)	+	+	-	-
27	Aspartic acid	2.82 (dd, *J* = 18.0, 4.5 Hz), 2.65 (d, 7.5)	+	+	-	-
28	Cucurbitacin E	6.08 (s), 4.65 (d, 8.0), 4.02 (m), 2.07 (m)	+	+	-	-
29	Formic acid	8.51 (s)	+	+	-	-
30	Gallic acid	7.05 (s)	-	-	+	+
31	Lycopene	6.17–6.68 (m), 5.95 (m), 5.11 (bs), 2.11 (bs), 2.00 (bs), 1.82 (s), 1.68 (s), 1.62 (s)	-	-	+	-
32	*β*-Carotene	1.49 (s), 1.62 (s), 1.72 (s), 2.03 (s), 6.17 (s), 6.19 (s)	-	-	+	-
33	Lutein	6.36 (d, *J* = 15.0 Hz), 6.25 (d, *J* = 15.5 Hz), 1.97 (s), 1.01 (s), 0.87 (s)	-	-	+	-
34	Violaxanthin	1.97 (s), 1.95 (s), 1.15 (s), 0.94 (s)	-	-	+	+
35	Zeaxanthin	1.97 (s), 1.98 (s), 1.77 (s), 1.07 (s)	-	-	+	+
36	Prolycopene	5.09 (m), 6.30 (m), 6.47 (m), 6.25 (d, *J* = 15.5 Hz), 1.64 (s), 1.56 (s), 1.97 (s)	-	-	+	-

Positive (+) and negative (-) signs denote present and absent, respectively.

**Table 2:** Yield and water loss of the watermelon water extracts presented as means ± standard deviation from six replicates. The different letters (a and b) within the column indicate significant difference (*p* < 0.05) between the two cultivars.

Sample	Crude Extract Weight (g)	Water Loss (g)
Red watermelon	8.45 ± 0.34 ^a^	381.86 ± 1.78 ^a^
Yellow watermelon	9.18 ± 0.07 ^b^	380.05 ± 1.88 ^a^

## Supplementary Materials

The following are available online, Figure S1: Stacking of representative ^1^H-NMR spectra of red watermelon in D_2_O (RW), yellow watermelon in D_2_O (YW), red watermelon in CDCl_3_ (RC), and yellow watermelon in CDCl_3_ (YC), Figure S2: DModX plot; the circle showing the position of the outlier from the principal component analysis (PCA) score plot, Figure S3: Hotelling’s T2 plot; the circle showing the position of the outlier from the principal component analysis (PCA) score plot, Figure S4: The orthogonal partial least squares discriminant analysis (OPLS-DA) score plot of ^1^H-NMR data representing red and yellow watermelon cultivars in D_2_O; R2X = 0.996, Q2X = 0.986, *n* = 6, Figure S5: The orthogonal partial least squares discriminant analysis (OPLS-DA) score plot of ^1^H-NMR data representing red and yellow watermelon cultivars in CDCl_3_; R2X = 0.981, Q2X = 0.953, *n* = 6,Figure S6: Permutation plot of orthogonal partial least squares discriminant analysis (OPLS-DA) model describing the Y-intercept of R2 (0.345) and Q2 (−0.754) for RW samples, Figure S7: Permutation plot of orthogonal partial least squares discriminant analysis (OPLS-DA) model describing the Y-intercept of R2 (0.333) and Q2 (−0.822) for YW samples, Figure S8: Permutation plot of orthogonal partial least squares discriminant analysis (OPLS-DA) model describing the Y-intercept of R2 (0.324) and Q2 (−0.795) for RC samples, Figure S9: Permutation plot of orthogonal partial least squares discriminant analysis (OPLS-DA) model describing the Y-intercept of R2 (0.349) and Q2 (−0.811) for YC samples, Table S1: CV-ANOVA validation test for orthogonal partial least squares discriminant analysis (OPLS-DA) score plot, Figure S10: Variable important in projection (VIP).
