# Supplementary material for: Metabolite Profiles of Red and Yellow Watermelon (Citrullus lanatus) Cultivars Using a 1H-NMR Metabolomics Approach
Source: Molecules. 2020 Jul 15;25(14):3235. doi: 10.3390/molecules25143235 (PMC7397335; doi:10.3390/molecules25143235)
Supplement: Supplementary file 1 [file molecules-25-03235-s001.pdf]

molecules

MDPI

Supplementary Materials

Metabolite Profiles of Red and Yellow Watermelon (*Citrullus lanatus*) Cultivars using a ^{1}H-NMR Metabolomics Approach

Fadzil Sulaiman [1], Amalina Ahmad Azam [1], Muhammad Safwan Ahamad Bustamam [1], Sharida Fakurazi [2], Faridah Abas [1], Yee Xuan Lee [1], Atira Adriana Ismail [1], Siti Munirah Mohd Faudzi [1] and Intan Safinar Ismail [1,*]

[1] Laboratory of Natural Products, Institute of Bioscience, Universiti Putra Malaysia, 43400 Serdang, Selangor, Malaysia; mfadzilsulaiman90@gmail.com (F.S.); amalina_azam@hotmail.com (A.A.A.); safwan.upm@gmail.com (M.S.A.B.); faridah_abas@upm.edu.my (F.A.); leeyeexuan0613@gmail.com (Y.X.L.); atiradriana@gmail.com (A.A.I.); sitimunirah@upm.edu.my (S.M.M.F.)

[2] Laboratory of Vaccines and Immunotherapeutics, Institute of Bioscience, Universiti Putra Malaysia, 43400 Serdang, Selangor, Malaysia; sharida@upm.edu.my

* Correspondence: safinar@upm.edu.my; Tel.: +60-3-9769 7492

Academic editor: Ewa Sikorska
Received: 20 May 2020; Accepted: 5 June 2020; Published: date

Abstract: Watermelon, a widely commercialized fruit, is famous for its thirst-quenching property. The broad range of cultivars, which give rise to distinct color and taste, can be attributed to the differences in their chemical profile, especially that of the carotenoids and volatile compounds. In order to understand this distribution properly, water extracts of red and yellow watermelon pulps with predominantly polar metabolites were subjected to proton nuclear magnetic resonance (^{1}H-NMR) analysis. Deuterium oxide (D_2O) and deuterated chloroform ($CDCl_3$) solvents were used to capture both polar and non-polar metabolites from the same sample. Thirty-six metabolites, of which six are carotenoids, were identified from the extracts. The clustering of the compounds was determined using unsupervised principal component analysis (PCA) and further grouping was achieved using supervised orthogonal partial least squares discriminant analysis (OPLS-DA). The presence of lycopene, *β*-carotene, lutein, and prolycopene in the red watermelon plays an important role in its differentiation from the yellow cultivar. A marked difference in metabolite distribution was observed between the NMR solvents used as evidenced from the PCA model. OPLS-DA and relative quantification of the metabolites, on the other hand, helped in uncovering the discriminating metabolites of the red and yellow watermelon cultivars from the same solvent system.

Keywords: ^{1}H-NMR; Citrullus lanatus; varieties; discriminants; carotenoids

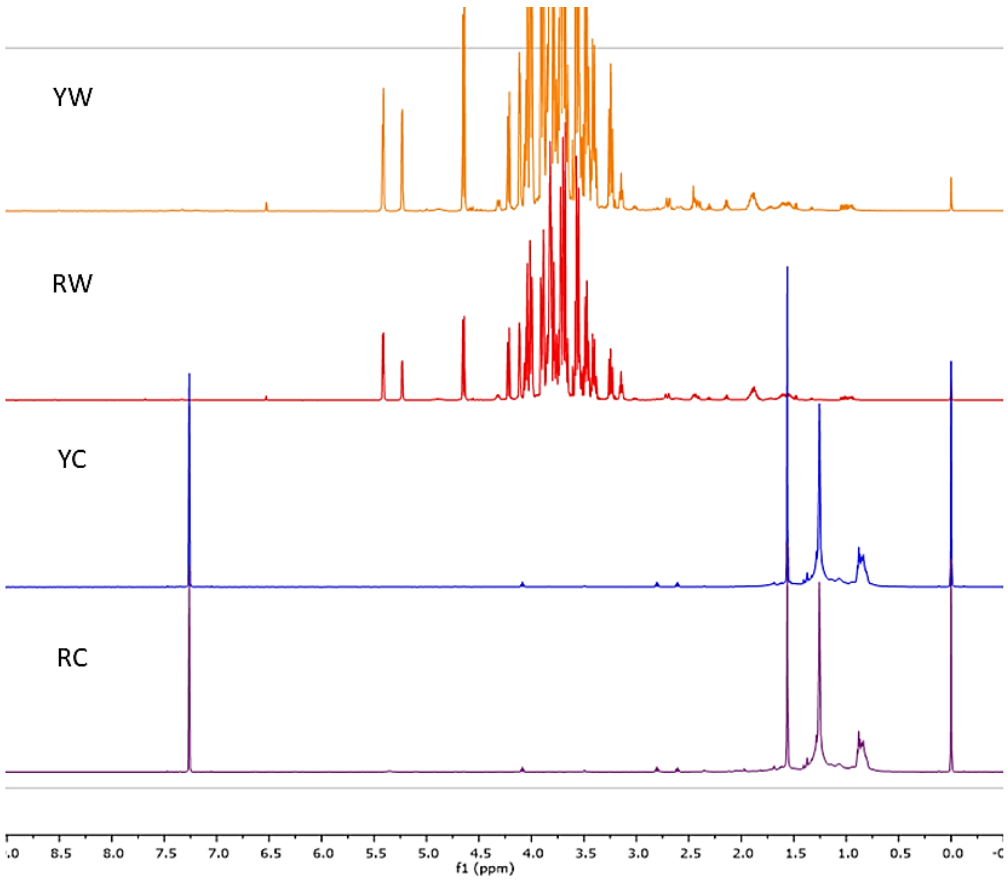

Figure S1: Stacking of representative ^{1}H-NMR spectra of red watermelon in D_2O (RW), yellow watermelon in D_2O (YW), red watermelon in $CDCl_3$ (RC), and yellow watermelon in $CDCl_3$ (YC).

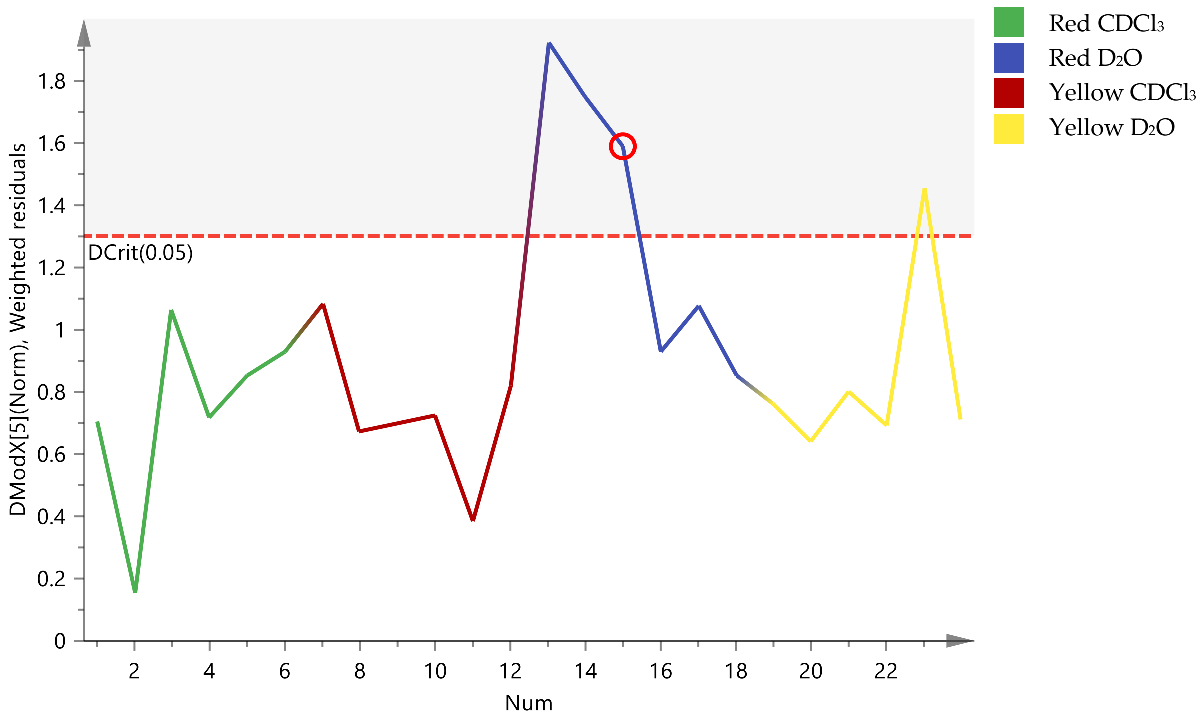

Figure S2: DModX plot; the circle showing the position of the outlier from the principal component analysis (PCA) score plot.

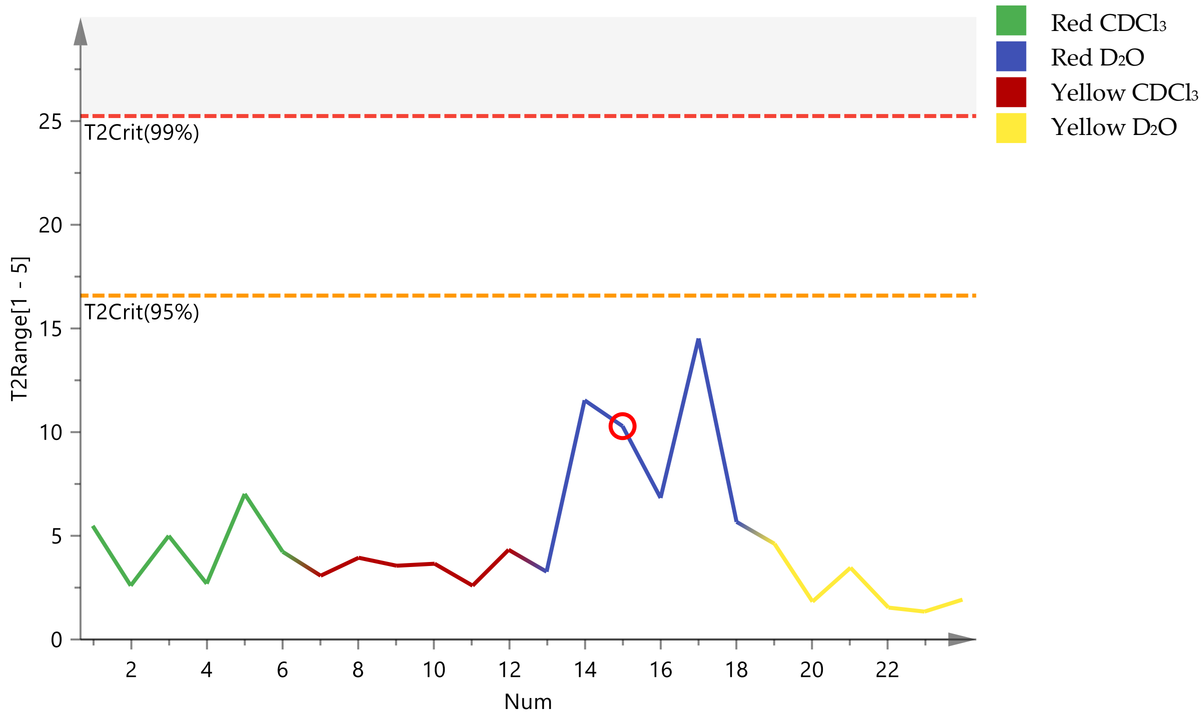

Figure S3: Hotelling's T2 plot; the circle showing the position of the outlier from the principal component analysis (PCA) score plot.

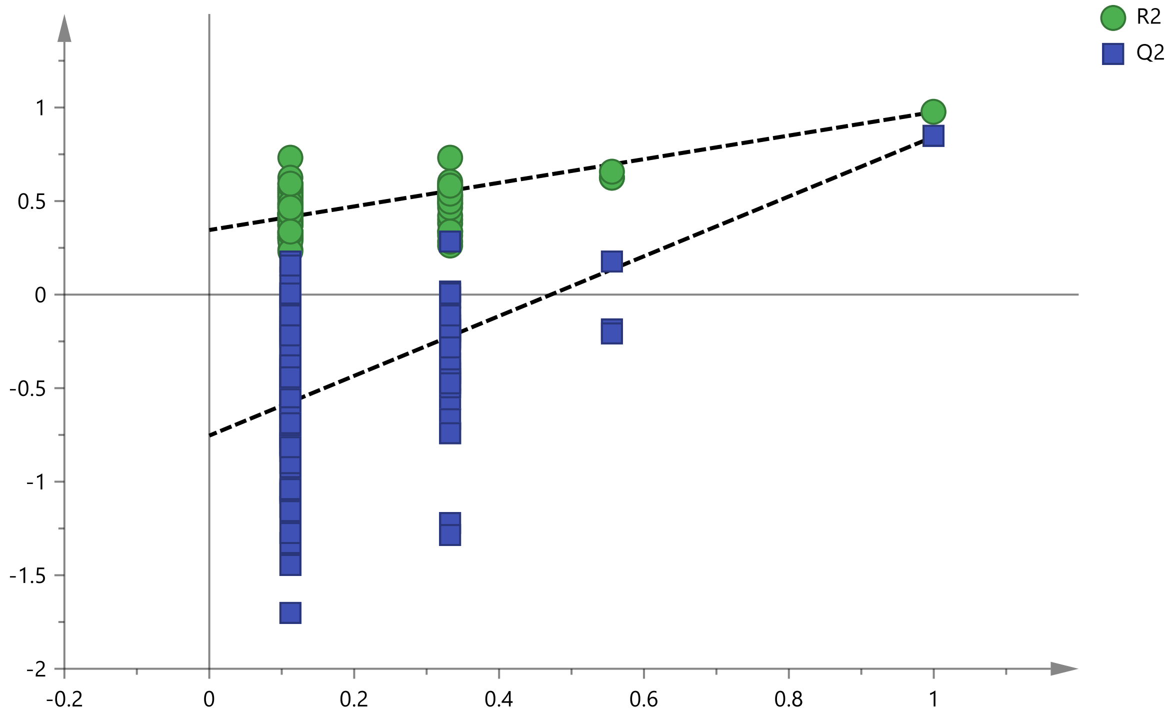

Figure S4: Permutation plot of orthogonal partial least squares discriminant analysis (OPLS-DA) model describing the Y-intercept of R2 (0.345) and Q2 (-0.754) for RW samples.

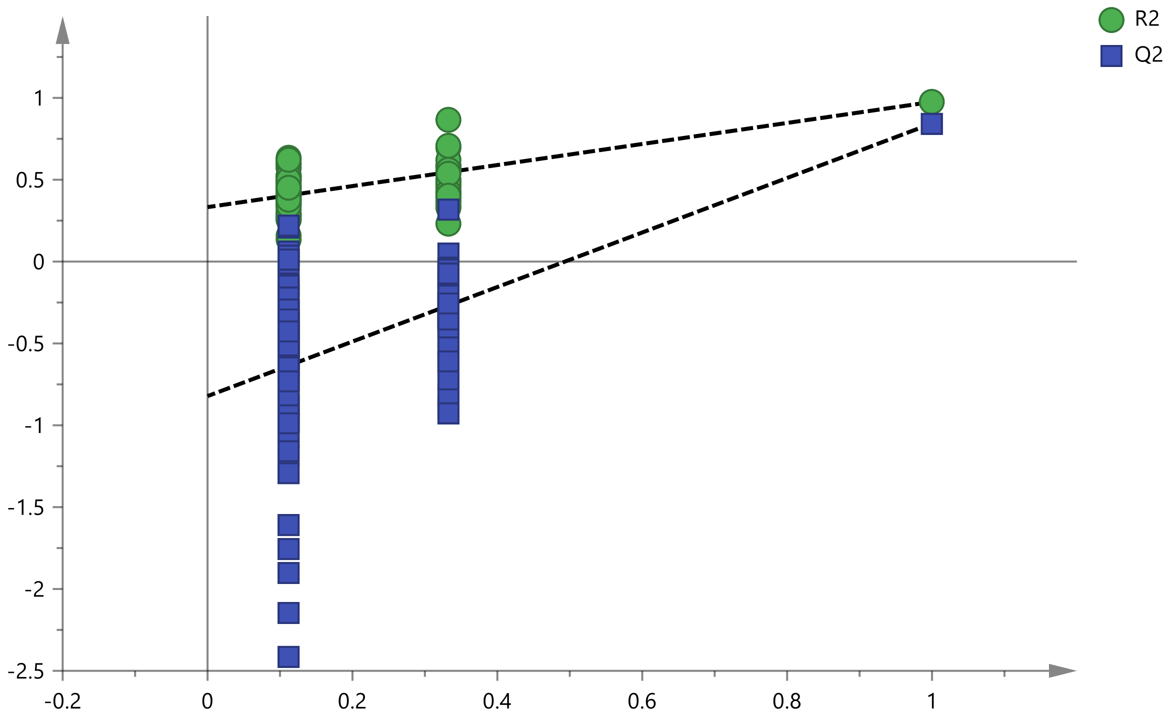

Figure S5: Permutation plot of orthogonal partial least squares discriminant analysis (OPLS-DA) model describing the Y-intercept of R2 (0.333) and Q2 (-0.822) for YW samples.

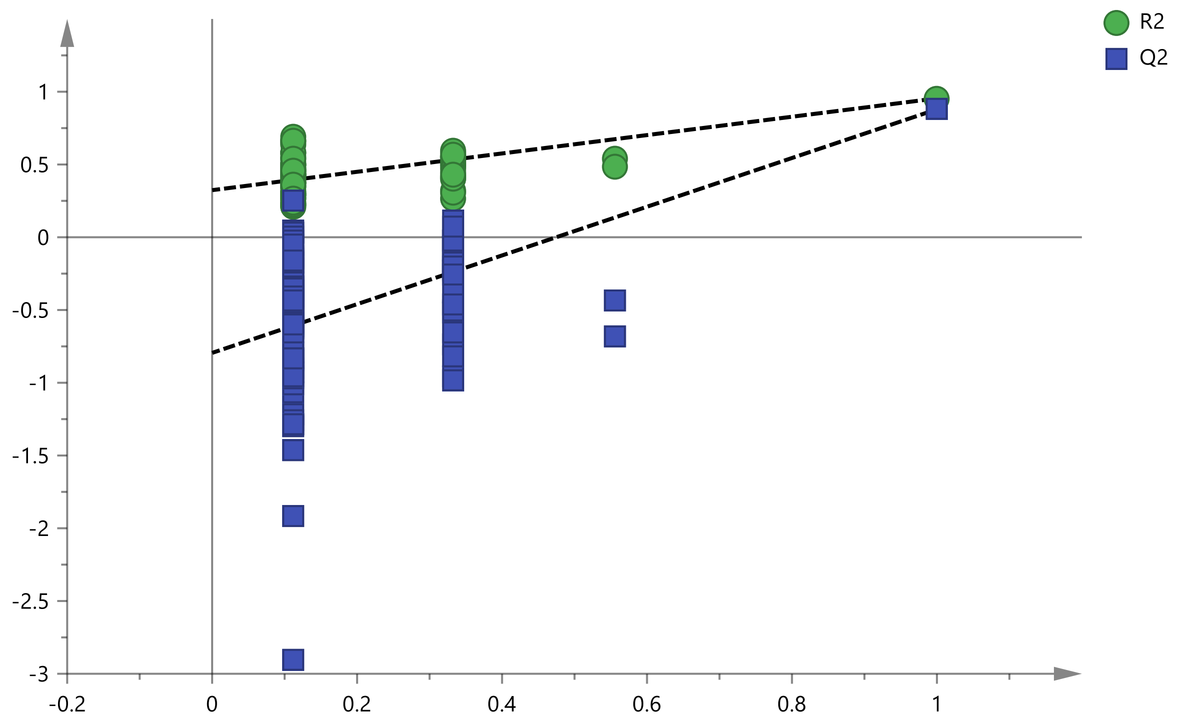

Figure S6: Permutation plot of orthogonal partial least squares discriminant analysis (OPLS-DA) model describing the Y-intercept of R2 (0.324) and Q2 (-0.795) for RC samples.

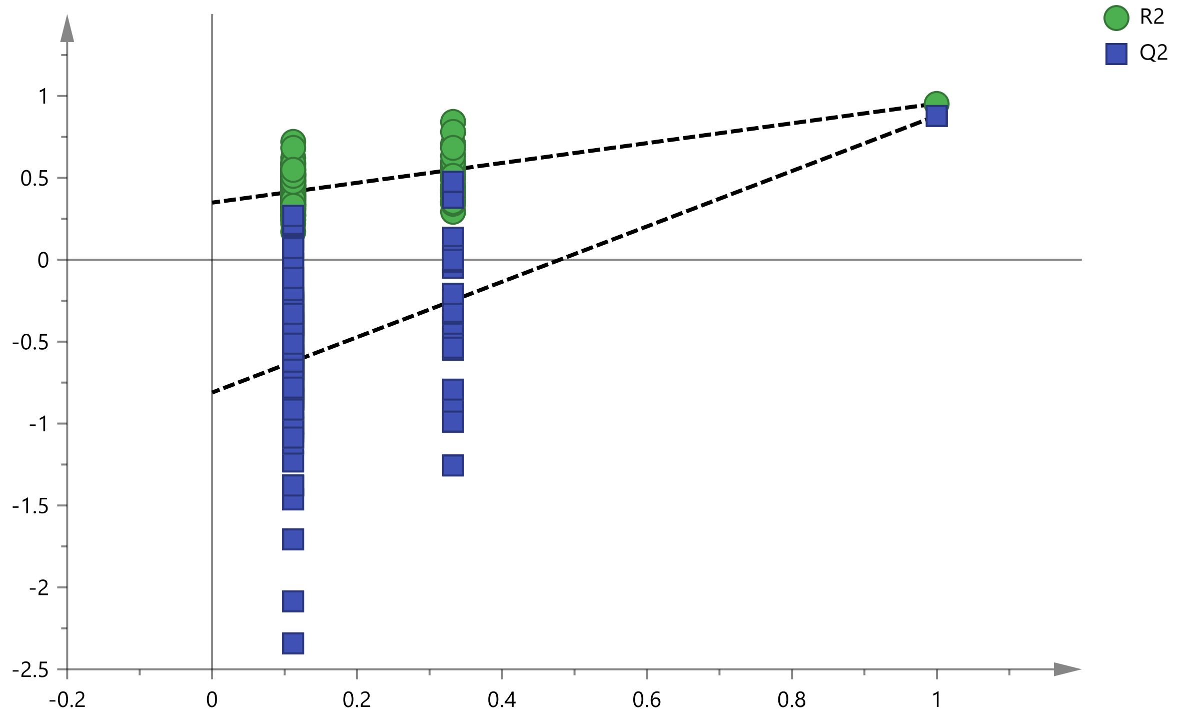

Figure S7: Permutation plot of orthogonal partial least squares discriminant analysis (OPLS-DA) model describing the Y-intercept of R2 (0.349) and Q2 (-0.811) for YC samples.

Table S1: CV-ANOVA validation test for orthogonal partial least square discriminant analysis (OPLS-DA) score plot.

M2(Untitled)	SS	DF	MS	F	p	SD
Total corr.	69	69	1			1
Regression	59.8708	30	1.99569	8.52563	1.21515e-009	1.41269
Residual	9.12919	39	0.234082			0.48382

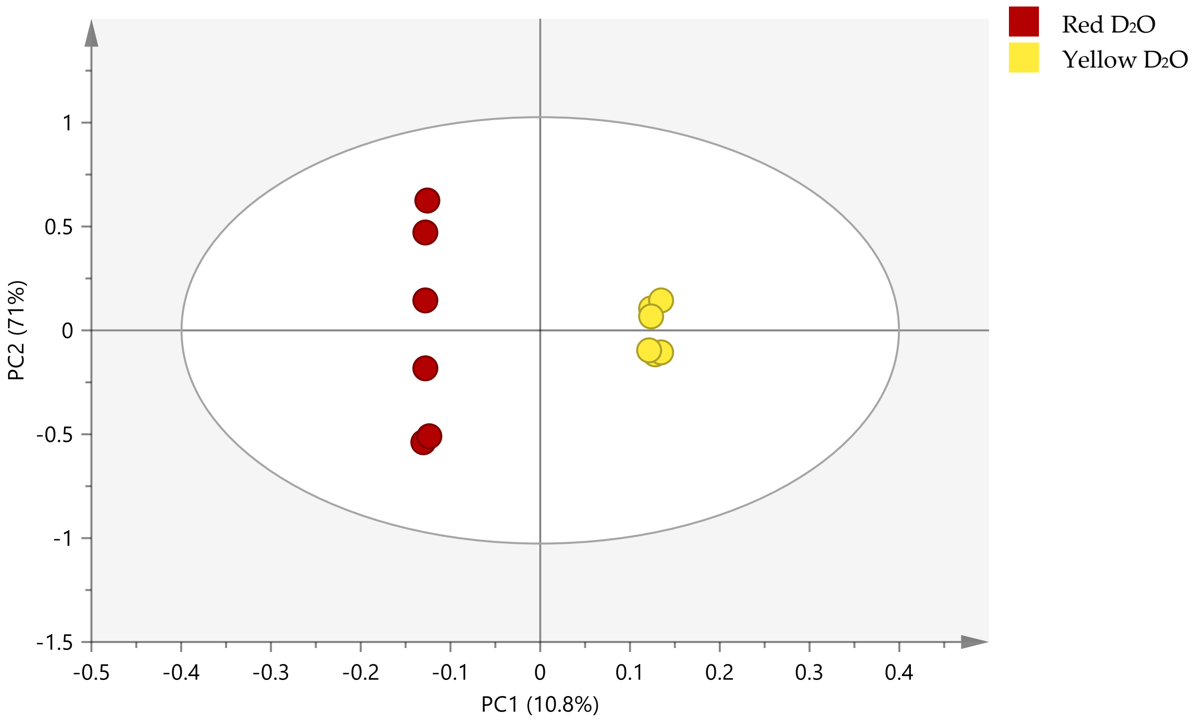

Figure S8. The orthogonal partial least squares discriminant analysis (OPLS-DA) score plot of ^{1}H-NMR data representing red and yellow watermelon cultivars in D_2O; R2X=0.996, Q2X = 0.986, n = 6.

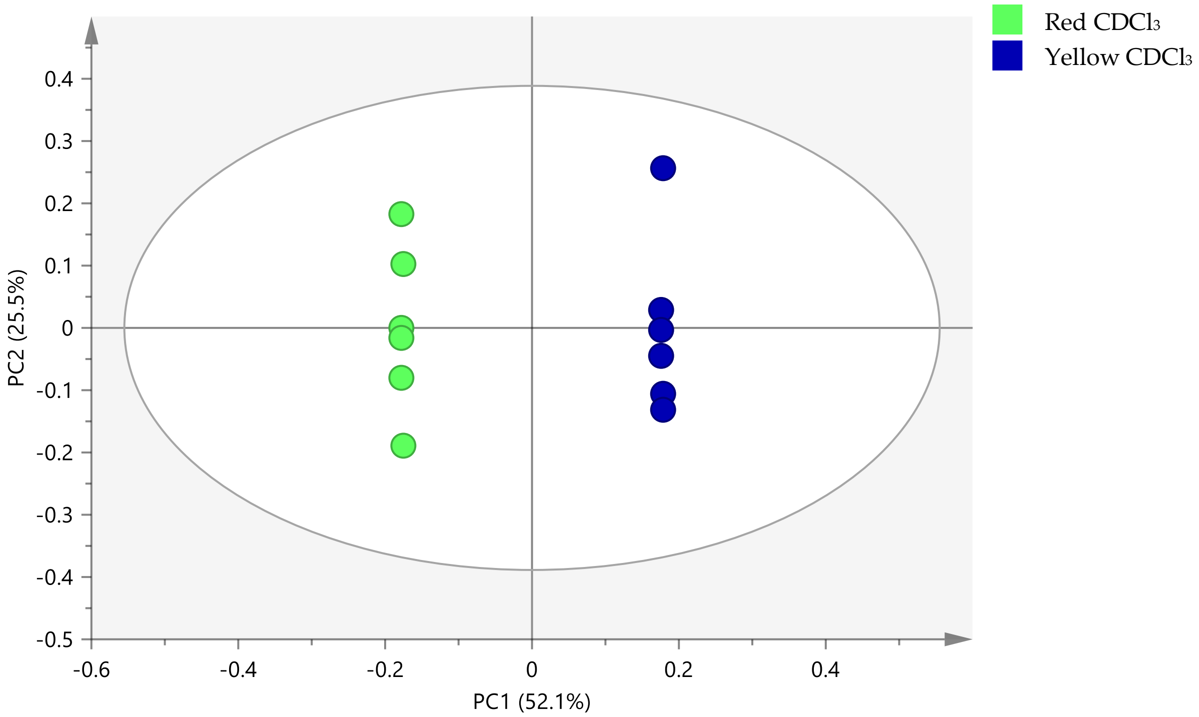

Figure S9. The orthogonal partial least squares discriminant analysis (OPLS-DA) score plot of ^{1}H-NMR data representing red and yellow watermelon cultivars in $CDCl_3$; R2X=0.981, Q2X = 0.953, n = 6.

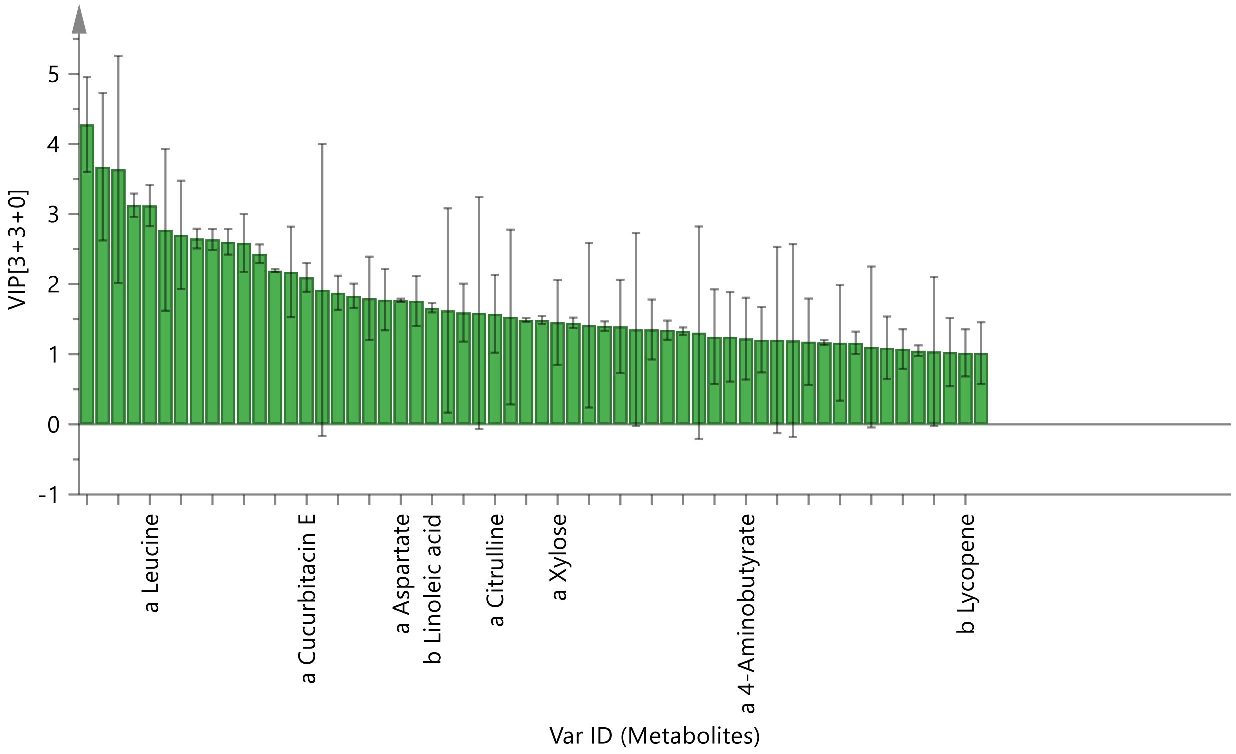

Figure S10: Variable important in projection (VIP).
